# The modulation of nicotinic acetylcholine receptors on the neuronal network oscillations in rat hippocampal CA3 area

**DOI:** 10.1038/srep09493

**Published:** 2015-03-26

**Authors:** Yang Wang, Zhan Wang, Jiangang Wang, Yali Wang, Zaineb Henderson, Xiaofang Wang, Xi Zhang, Jinggui Song, Chengbiao Lu

**Affiliations:** 1 Key Laboratory for the Brain Research of Henan Province, Xinxiang Medical University, Henan Province, Henan PR. China; 2 Institute of Membrane and System Biology, University of Leeds, Leeds, England; 3 Psychiatric Hospital of Henan Province, 2nd Affiliated Hospital of Xinxiang Medical University

## Abstract

γ oscillations are associated with higher brain functions such as memory, perception and consciousness. Disruption of γ oscillations occur in various neuro-psychological disorders such as schizophrenia. Nicotinic acetylcholine receptors (nAChR) are highly expressed in the hippocampus, however, little is known about the role on hippocampal persistent γ oscillation. This study examined the effects of nicotine and selective nAChR agonists and antagonists on kainate-induced persistent γ oscillation in rat hippocampal slices. Nicotine enhanced γ oscillation at concentrations of 0.1–10 μM, but reduced it at a higher concentration of 100 μM. The enhancement on γ oscillation can be best mimicked by co-application of α4β2- and α7- nAChR agonist and reduced by a combination of nAChR antagonists, DhβE and MLA. However, these nAChR antagonists failed to block the suppressing role of nicotine on γ. Furthermore, we found that the NMDA receptor antagonist D-AP5 completely blocked the effect of nicotine. These results demonstrate that nicotine modulates γ oscillations via α7 and α4β2 nAChR as well as NMDA activation, suggesting that nAChR activation may have a therapeutic role for the clinical disorder such as schizophrenia, which is known to have impaired γ oscillation and hypo-NMDA receptor function.

Fast network oscillations in the γ frequency band (30–80 Hz; γ oscillation) are associated with brain function such as attention, working memory and sensory information processing^1^,^2^,^3^,^4^. The parvalbumin (PV)-expressing interneurons provide strong inhibitory input to pyramidal neurons and play a critical role in the synchronization of neuronal firing within the network, a basic mechanism for the generation of γ oscillations^5^.

Cholinergic input modulates hippocampal network oscillations^6^,^7^,^8^. The muscarinic acetylcholine receptor (mAChR) agonist, carbachol, induces theta and γ oscillations in hippocampal slices *in vitro*^9^,^10^,^11^. The mAChR antagonists reduce γ power, decrease theta oscillation frequency and weaken interaction between γ and theta oscillations^12^.

Recently, nicotinic acetylcholine receptor (nAChR) agonist, nicotine, has been reported to induce theta activity in the hippocampus^13^ and augments stimulation-induced hippocampal theta oscillation via activation of alpha7 acetylcholine receptors^6^. Relatively little is known about the modulation of nAChR on fast network oscillations such as γ oscillation. Although nicotine is not able to induce γ oscillation, it appears to enhance auditory evoked γ oscillations^14^, but the mechanism of nicotinic modulation of γ oscillations remains largely unknown.

α7 and α4β2 nAChRs are two subunits of nAChRs commonly expressed in the brain. α7 nAChRs are located on glutamatergic and GABAergic terminals and modulate the release of glutamate and GABA^15^,^16^,^17^. α4β2 nAChRs are expressed in GABAergic interneurons and modulate GABA release^16^,^18^,^19^. It has been recently reported that α4β2 nAChRs expressed in glutamatergic terminals regulate glutamate release in prefrontal cortex^20^. It is expected that nicotine may activate these receptors and modulate γ oscillations^14^,^21^.

The patients with the neuro-psychological disorders such as schizophrenia are associated with disruption of γ oscillations^22^,^23^, reflecting the dysfunction in sensory information processing and cognitive control in these patients^24^,^25^. Patients with schizophrenia may be associated with NMDAR hypofunction, as blockade of MDA receptor mimics schizophrenic-like symptoms in both humans and animal model of the disease^26^,^27^, and induces aberrant γ oscillations^28^,^29^,^30^. Interestingly, nicotine enhances NMDA-mediated current^31^, ameliorates NMDA receptor antagonist-induced deficits in contextual fear conditioning through α4β2 nAChR in the hippocampus^32^ and enhances NMDA cognitive circuits via α7 nAChR activation in dorsolateral prefrontal cortex^33^. These studies indicate that nicotine enhances NMDA receptor function through activation of specific nAChR subunits. Whether NMDA receptor is involved in the modulation of nicotine on γ oscillations is unknown, although the pharmacologically-induced persistent γ oscillations do not require NMDA receptor activation^34^,^35^.

Therefore, this study aimed to investigate the roles of nAChR activation on γ oscillations, clarify the nAChR subunit-specific involvement and determine whether NMDA receptor is involved. We chose the commonly-used model of γ oscillations, which can be stable for hours, necessity for the investigation of the roles of various nAChR antagonists and agonists on γ. We demonstrated that low concentrations of nicotine enhanced kainate-induced persistent γ oscillation through α4β2 and α7 nAChRs as well as NMDA receptor activation and that higher concentration of nicotine reduced γ through an NMDA receptor-dependent effect. This study suggests that tonic activation of nAChR modulates hippocampal network oscillations with a positive and negative consequence depending on the concentration of nicotine, thus manipulation of the strength of nAChR activation will be crucial for the improving cognitive function in pathological conditions such as schizophrenia, which is known to have impaired γ and NMDA receptor hypofunction.

## Methods

### Animals

All experimental protocols were approved by the Animal Experimentation Ethics Committees of Xinxiang Medical University and Leeds University, and all efforts were made to minimize animal suffering and reduce the number of animals used. All experiments were performed in accordance with the guidelines of the Animal Care and Use Committee of Xinxiang Medical University and Leeds University. Electrophysiological studies were performed on hippocampal slices prepared from Wistar rats (male, 4–5 week-old). For electrophysiology, the animals were anaesthetised by intraperitoneal injection of Sagatal (sodium pentobarbitone, 100 mg kg^−1^, Rhône Mérieux Ltd, Harlow, UK). When all pedal reflexes were abolished, the animals were perfused intracardially with chilled (5°C), oxygenated artificial cerebrospinal fluid (ACSF) in which the sodium chloride had been replaced by iso-osmotic sucrose. This ACSF (305 mosmol l^−1^) contained (in mM): 225 sucrose, 3 KCl, 1.25 NaH_2_PO_4_, 24 NaHCO_3_, 6 MgSO_4_, 0.5 CaCl_2_ and 10 glucose. For extracellular field recording, the hippocampal horizontal slices (450 μm) of rat brain were cut at 4–5°C in the sucrose-ACSF, using a Leica VT1000S vibratome (Leica Microsystems UK, Milton Keynes, UK).

### Electrophysiological recording, data acquisition and analysis

For extracellular field recordings, the two hippocampal slices were transferred to an interface recording chamber. The slices were maintained at a temperature of 32°C and at the interface between ACSF and warm humidified carbogen gas (95% O_2_-5% CO_2_). The ACSF contained (in mM): 126 NaCl, 3 KCl, 1.25 NaH_2_PO_4_, 24 NaHCO_3_, 2 MgSO_4_, 2 CaCl_2_ and 10 Glucose. The slices were allowed to equilibrate in this medium for 1 h prior to recording. Both channels of an Axoprobe 1A amplifier (Axon Instruments, Union City, CA, USA) were employed for extracellular field recordings, which were made using glass microelectrodes containing ACSF (resistance 2–5 MΩ). Data were band-pass filtered online between 0.5 Hz and 2 kHz using the Axoprobe amplifier and a Neurolog system NL106 AC/DC amplifier (Digitimer Ltd, Welwyn Garden City, UK). The data were digitized at a sample rate of 5–10 kHz using a CED 1401 plus ADC board (Digitimer Ltd). Electrical interference from the mains supply was eliminated from extracellular recordings online with the use of 50 Hz noise eliminators (HumBug; Digitimer Ltd).

Data were analyzed off-line using software from Spike 2 (CED, Cambridge, UK). Power spectra were generated to provide a quantitative measure of the frequency components in a stretch of recording, where power, a quantitative measure of the oscillation strength, was plotted against the respective frequency. Power spectra were constructed for 30–60 s epochs of extracellular field recordings using a fast Fourier transform algorithm provided by Spike2. The parameters used for measuring the oscillatory activity in the slice were peak frequency (Hz) and area power ( μV^2^). In the current study, area power was equivalent to the computed area under the power spectrum between the frequencies of 20 and 60 Hz.

All statistical tests were performed using SigmaStat software (SPSS Inc., California, USA). Results are expressed as mean ± standard error of mean, unless indicated otherwise. Statistical significance for comparison between two groups or among three groups was determined using tests described in the text or in the figure legends, as appropriate. Measures were considered statistically significant, if *P* < 0.05.

### Drugs used for electrophysiology

All standard reagents, except where indicated, were obtained either from Sigma-Aldrich (UK) or VWR International (Lutterworth, UK). D-(–)-2-amino-5-phosphonopentanoic acid (D-AP5), bicuculline methochloride, SR 95531 hydrobromide (GABAzine) and 2,3,-dioxo-6-nitro-1,2,3,4-tetrahydrobenzo[f]quinoxaline-7-sulphonamide (NBQX) were purchased from Tocris Cookson Ltd (Bristol, UK). Kainate,atropine sulphate, choline, dihydro-β-erythroidine (DHβE), methyllycaconitine (MLA), nicotine sulphate, PNU282987, RJR2403 and agents for the ACSF solution were obtained from Sigma-Aldrich (UK). Stock solutions, at 10^3^ of the working concentration, were made up in water, except for NBQX which was dissolved in dimethylsulphoxide and stored in individual aliquots at −20°C. Working solutions were prepared freshly on the day of the experiment.

## Results

### Nicotine increased γ frequency oscillations

**Figure 1:**
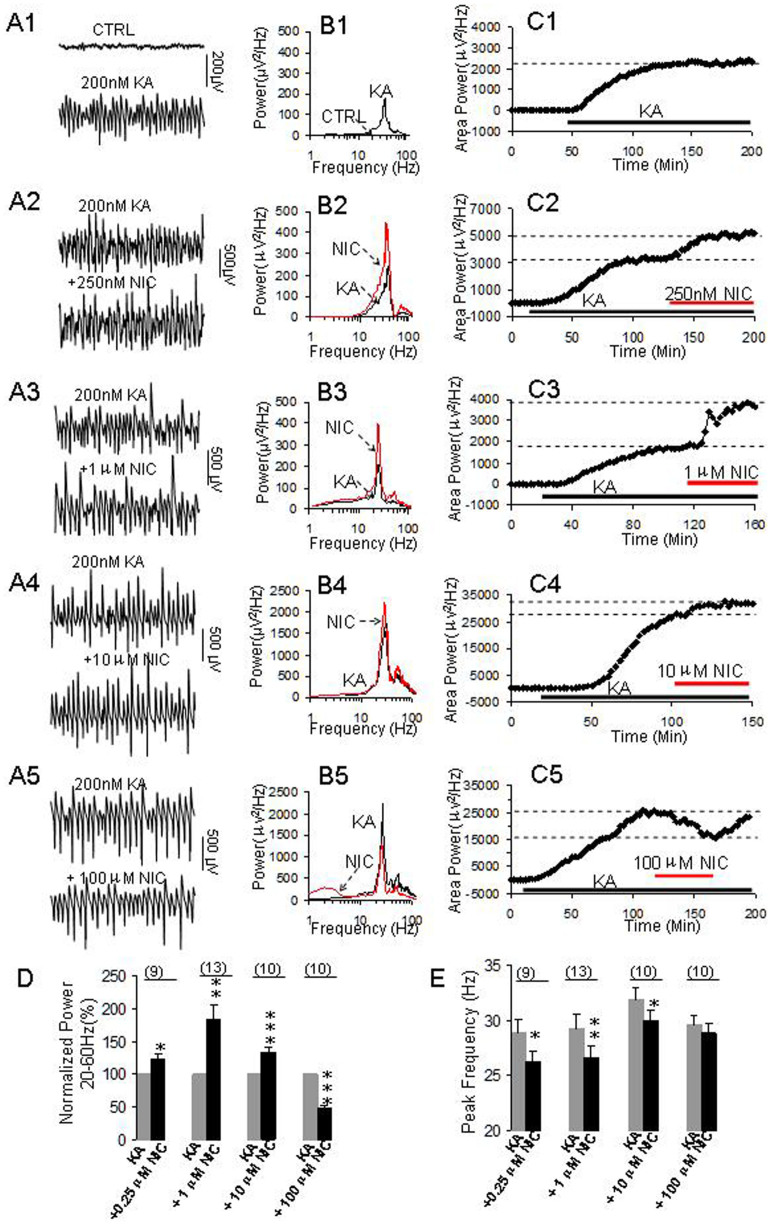
The effects of nicotine on γ oscillations. (A1–C1) KA-induced γ oscillation. (A1): Representative traces of extracellular recordings in hippocampal CA3 before and after KA application; The 1-second waveforms were taken from the steady states before and after application of KA. (B1): The power spectra of the field potentials before and after application of KA; (C1): The time course shows the changes of γ power before and after application of KA. (A2–A5) Representative extracellular recordings of field potentials before and after application of nicotine at 0.25 μM (A2), 1 μM (A3), 10 μM (A4) and 100 μM (A5). (B2–B5) Power spectra of field potentials before and after application of nicotine at 0.25 μM (B2), 1 μM (B3), 10 μM (B4) and 100 μM (B5); (C2–C5) The time courses showing the changes of γ power before and after application of nicotine at 0.25 μM (C2); 1 μM (C3), 10 μM (C4) and 100 μM (C5). (D): Bar graph summarizes the percent changes in γ power before and after application of various concentrations of nicotine. Gray bar: Normalized γ power in control (100%, KA alone). Black bars: The percent changes in γ powers after application of various concentrations of nicotine. *p < 0.05, **p < 0.01, ***p < 0.001, compared with control, one way RM ANOVA, n = 9, 13, 10, 10 for 0.25 μM, 1 μM, 10 μM and 100 μM nicotine, respectively. (E): Bar graph summarizes the changes in peak frequency of γ oscillations before and after application of various concentrations of nicotine. Gray bars: Control peak frequency (KA alone), Black bars: The peak frequency after application of various concentrations of nicotine (*p < 0.05, **p < 0.01, compared with control, one way RM ANOVA).

Kainate (KA, 200 nM) induced persistent γ oscillation (20–60 Hz) in rat hippocampal CA3 area. γ oscillation usually takes approximately 1 to 2 hours to achieve steady-state and would last for at least three hours (Fig. 1A1, B1, C1), which is in agreement with previous studies^35^,^36^,^37^. γ oscillations can be blocked by the AMPA/kainate receptor antagonist, NBQX (20 μM), or the GABA_A_ receptor antagonist, bicuculline (20 μM) (n = 5, data not shown), confirming that these oscillations are mediated by excitatory and inhibitory neurotransmission.

When γ oscillations reached a steady state, various concentrations of nicotine (0.1–100 μM) were administered with ACSF. At 0.25 μM, nicotine caused a 23 ± 7% increase in the γ power (*p < *0.05*, compared with control, one-way repeated measures ANOVA, n = 9, Fig. 1A2–C2, D). At 1 μM, nicotine caused a large increase of 83 ± 21% in γ power (**p < *0.01*, n = 13, Fig. 1A3–C3, D). At a higher concentration of 10 μM, nicotine caused a 32 ± 7% increase in γ power (***p < *0.001*, n = 10, Fig. 1A4–C4, D). When the concentration further increased to 100 μM, nicotine caused a reversible reduction (49 ± 4%) in γ power (***p < 0.001, n = 10, Fig. 1A5–C5, D). Our results demonstrated that nicotine enhanced persistent γ oscillations at a relative low concentration but decreased it at a higher concentration in the hippocampal CA3 area.

The increase in γ power was associated with a slight decrease in peak frequency after applications of nicotine. On average, the peak frequency was decreased 2.6 ± 0.4 Hz (*p < 0.05, n = 9, one way RM ANOVA, Fig. 1E), 2.7 ± 0.4 Hz (**p < 0.01, n = 13) and 2.0 ± 0.5 Hz (*p < 0.05, n = 10) for applications of 0.25 μM, 1 μM and 10 μM nicotine, respectively. However, 100 μM nicotine had no significant effect on the peak frequency (*p > 0.05*, n = 10).

### The roles of selective nAChR agonists on γ power

**Figure 2:**
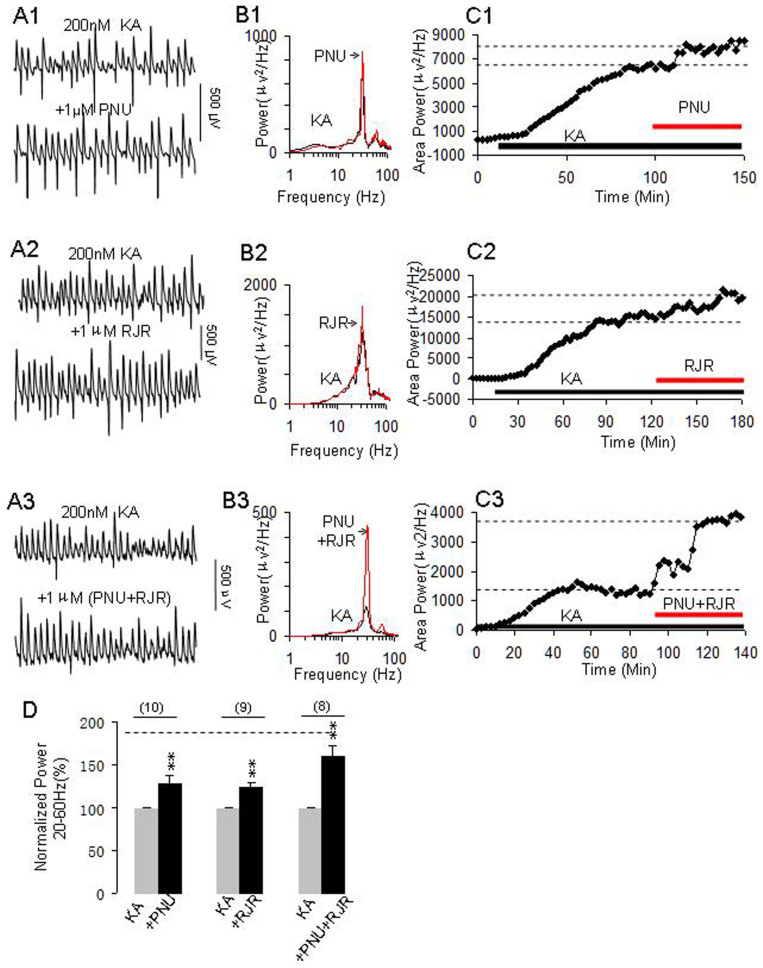
The effects of selective nAChR agonists on γ oscillations. (A1–A3) Representative extracellular recordings of KA-induced field potentials before and after application of α7 nAChR agonist PNU282987 (PNU, 1 μM) (A1), α4β2 nAChR agonist RJR2403 (RJR, 1 μM) (A2) and PNU + RJR (A3). The 1-second waveforms were taken from the steady states under various conditions. (B1–B3) The power spectra of KA-induced field potentials before and after applications of PNU (B1), RJR (B2) and PNU + RJR (B3). (C1–C3) The time course shows the changes in γ power before and after application of PNU (C1), RJR (C2) and PNU + RJR (C3). (D): Bar graph shows the effects of PNU, RJR or PNU + RJR on γ power. Gray bars: Normalized γ power in control (100%, KA alone), Black bars: percent changes in γ powers after application of PNU (n = 10), RJR (n = 9) or PNU + RJR (n = 8). **p < 0.01, compared with control, one way RM ANOVA. The dashed horizontal line located at the top of the graph D indicates the level of percentage change on γ oscillations induced by nicotine (1 μM) alone.

To determine which nAChR subunits play a role on γ enhancement of nicotine, we further tested the effects of the selective α7 nAChR agonist PNU282987 or the α4β2 nAChR agonist RJR2403 alone or in the combination on γ oscillations. Application of PNU282987 (1 μM) or RJR2403 (1 μM) alone enhanced γ oscillation as shown in Fig. 2A1–C1, A2–C2 by representative experiments. The combination of two agonists dramatically enhanced γ power (Fig. 2A3–C3). On average, the percent increase in γ-power was 28 ± 9%, 25 ± 6%, and 61 ± 13% for PNU282987 (n = 10), RJR2403 (n = 9) and PNU282987 + RJR2403 (n = 8), respectively. Compared with control, these changes are all of statistical significance (*p < 0.01, one way RM ANOVA, Fig. 2D).

### Roles of selective nAChR antagonists on nicotine's role

To determine the involvement of specific nAChR subunits on nicotine's role on γ oscillation, the hippocampal slices were pretreated with the selective α4β2 nAChR antagonist DhβE, the selective α7 nAChR antagonist MLA or a combination of both antagonists to see whether these antagonists can preclude nicotine's effects on γ. The hippocampal slices were pretreated with DhβE (0.2 μM) or MLA (0.2 μM) or both for 20 min before KA application. The antagonists either alone or in a combination did not affect γ development nor γ power, as the time for reaching a steady state of γ oscillations were not significantly different between control (KA alone, 86 ± 3 min, n = 25) and the pretreatment of MLA (83 ± 6 min, n = 6) or DhβE (77 ± 3 min, n = 6) or a combination of MLA and DhβE (82 ± 2 min, n = 7) and the γ powers were not significantly different between control (KA alone, 6694 ± 1226 μV^2^, n = 25) and the pretreatment of MLA (4257 ± 1762 μV^2^, n = 6) or DhβE (6076 ± 2001 μV^2^, n = 6) or a combination of MLA and DhβE (3558 ± 2145 μV^2^, n = 7).

**Figure 3:**
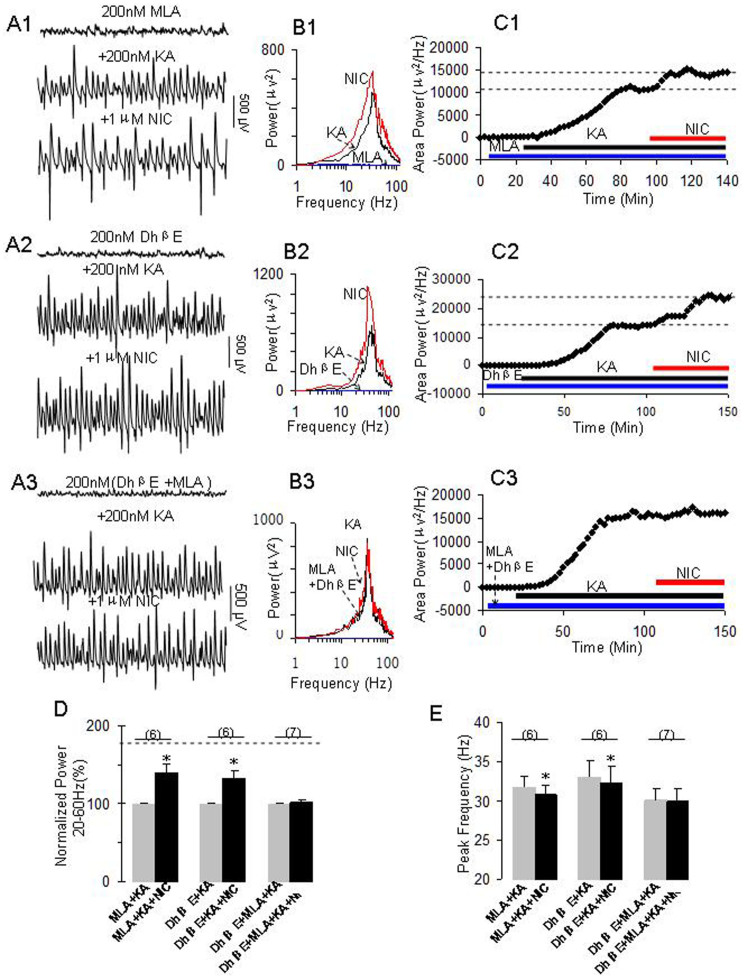
The effects of selective nAChR antagonists on nicotine's role on γ oscillations. (A1): Representative extracellular recordings in the presence of MLA (200 nM), MLA + KA (200 nM) and MLA + KA + NIC (1 μM). The 1-second waveforms were taken from the steady states under various conditions. (B1): The power spectra of field potentials corresponding to the conditions shown in A1. (C1): The time course shows the changes in γ power before and after application of NIC in the presence of MLA. (A2): Representative extracellular recordings in the presence of DhβE (200 nM), DhβE + KA and DhβE + KA + NIC. (B2): The power spectra of field potentials corresponding to the conditions shown in A2. (C2): The time course shows the changes in γ power before and after application of NIC in the presence of DhβE. (A3): Representative extracellular recordings in the presence of DhβE + MLA, DhβE + MLA + KA and DhβE + MLA + KA + NIC. (B3): The power spectra of field potentials corresponding to the conditions shown in A3. (C3): The time course shows the changes in γ power before and after application of NIC in the presence of DhβE + MLA. (D). The bar graph summarizes the percent changes in γ power before and after application of nicotine in the presence of various nAChR antagonists. Gray bars: Normalized control γ powers for MLA + KA, DhβE + KA or DhβE + MLA + KA; Black bars: percent changes in γ powers after application of nicotine in the presence of MLA + KA, DhβE + KA or DhβE + MLA + KA (**p < 0.01, compared with their own controls, one-way RM ANOVA). (E): Bar graph summarizes the changes in peak frequency in γ oscillations before and after application of nicotine in the presence of nAChR antagonists alone or combined. Gray bars: The peak frequencies before application of nicotine in the presence of MLA + KA, DhβE + KA or DhβE + MLA + KA. Black bars: The peak frequencies after application of nicotine in the presence of MLA + KA, DhβE + KA or DhβE + MLA + KA (*p < 0.05, one-way RM ANOVA).

After the steady state of γ oscillations was reached in the presence of these nAChR antagonists, nicotine (1 μM) was applied. Our results showed that MLA (Fig. 3A1–C1) or DhβE (Fig. 3A2–C2) partially reduced nicotinic enhancement on γ power, but a combination of both antagonists blocked the nicotinic effect (Fig. 3A3–C3). On average, nicotine caused 40 ± 11% (*p < 0.05, one way RM ANOVA, n = 6), 33 ± 10% (*p < 0.05, n = 6) and 1 ± 3% (*p > 0.05*, n = 7) increase in γ power for the pretreatment of MLA, DhβE and MLA + DhβE, respectively (Fig. 3D). Two way RM ANOVA also revealed that there was a significant interaction between nAChR antagonists and nicotine for the pretreatment of MLA + DhβE (*p < 0.01) and DhβE (*p < 0.05) but not for MLA (p > 0.05). These results indicate that MLA + DhβE pretreatment effectively blocks nicotine-induced increase in γ power.

In terms of peak frequency, nAChR antagonist alone partially reduced the effect of nicotine on peak frequency of the oscillations; a combination of both antagonists blocked the decrease of peak frequency induced by nicotine. On average,nicotine caused 1.0 ± 0.3 Hz (*p < 0.05, one-way RM ANOVA, n = 6), 0.7 ± 0.2 Hz (*p < 0.05, n = 6) and 0.1 ± 0.3 Hz (p > 0.05, n = 7) decrease in the peak frequency for the pretreatment of MLA, DhβE or MLA + DhβE, respectively (Fig. 3E). Two-way RM ANOVA also revealed that there was a significant interaction between nAChR antagonists and nicotine for the pretreatment of MLA + DhβE (***p < 0.001), MLA (*p < 0.05) and DhβE (**p < 0.01), indicating that these antagonists either alone or in a combination blocked the nicotine-induced changes in peak frequency.

In a different set of experiments (n = 10), we also investigated the effects of these antagonists on nicotine's role in the conditions of these antagonists being applied when γ power reached a steady state. Similar to the pretreatment of these antagonists, only a combination of both α7 nAChR and α4β2 nAChR antagonists can block nicotine role (data not shown).

### Selective nAChR antagonists blocked nicotine-mediated enhancing role but not suppression effect on γ oscillations

**Figure 4:**
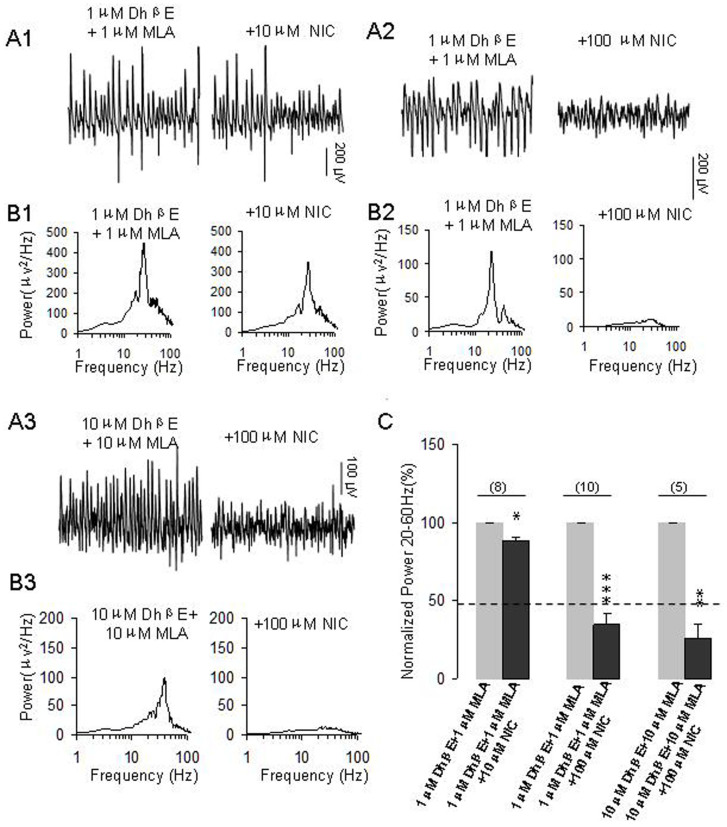
The effects of pretreatment of nAChR antagonists on the roles of higher concentrations of nicotine on γ oscillations. (A1): Representative extracellular recordings of field potentials induced by KA (200 nM) in the presence of DhβE (1 μM) + MLA (1 μM) and DhβE + MLA + NIC (10 μM). (B1): The power spectra of field potentials corresponding to the conditions shown in A1. (A2): Representative extracellular recordings of field potentials induced by KA (200 nM) in the presence of DhβE (1 μM) + MLA (1 μM) and DhβE + MLA + NIC (100 μM). (B2): The power spectra of field potentials corresponding to the conditions shown in A2. (A3): Representative extracellular recordings of field potentials induced by KA (200 nM) in the presence of DhβE (10 μM), MLA (10 μM) and DhβE + MLA + NIC (100 μM). (B3): The power spectra of field potentials corresponding to the conditions shown in A3. (C): Bar graph summarizes the percent changes in γ power before and after application of nicotine at10 μM and 100 μM in the pretreatment of DhβE + MLA (1–10 μM for both). Gray bars: The percent changes in γ power in the pretreatment of DhβE + MLA. Black bars: The percent changes in γ power after application of nicotine in the pretreatment of DhβE + MLA (*p < 0.05, **p < 0.01, ***p < 0.001, compared with control, one way RM ANOVA).

We then tested whether the combined antagonists affect the role of nicotine at higher concentrations. In the presence of MLA + DhβE, 10 μM nicotine caused 11.7 ± 2.2% decrease on γ power (*p < 0.05, compared with control, n = 8, Fig. 4A1, B1, C). These results suggest that nAChR antagonists blocked the nicotine-mediated enhancing role on γ and exposed a small, inhibitory effect of 10 μM nicotine on γ oscillation.

Furthermore, we tested the effects of co-application of MLA and DhβE on the role of 100 μM nicotine on γ. Our results showed that these antagonists did not affect the γ power per se, but enhanced nicotine-mediated suppression of γ (Fig. 4 A2, B2). In the presence of DhβE + MLA, 100 μM nicotine caused a 70 ± 5% decrease on γ power (***p < 0.001, n = 10, Fig. 4C). Compared with γ power in the presence of 100 μM nicotine alone (the dashed line shown in Fig. 4C), such a change was of statistical significance (*p < 0.01, two way RM ANOVA). These results indicate that blockage of nAChR enhanced nicotine-mediated suppression on γ power.

In the presence of DhβE + MLA, further application of 10 μM or 100 μM nicotine (in different set of experiments) did not alter peak frequency. On average, 10 μM and 100 μM nicotine caused 1 ± 1 Hz (n = 8) and 0.4 ± 1 Hz (n = 10) reduction of peak frequency, respectively (*p* > 0.05, compared with the control).

The co-application of DhβE and MLA both at low micromolar range failed to block the effect of 100 μM nicotine, the concentration of both nAChR antagonists was increased to 10 μM and their effects on the role of nicotine on γ were further tested. Co-application of DhβE and MLA both at 10 μM failed to block nicotine-mediated suppression of γ power (Fig. 4A3, B3, C, n = 5), they rather enhanced nicotine-mediated suppression of γ. On average, in the presence of DhβE + MLA, 100 μM nicotine caused 74 ± 9% decrease on γ power (*p < 0.05, compared with control). Compared with application of 100 μM nicotine alone, this change was of a statistical significance (*p < 0.01, two-way RM ANOVA).

### NMDA receptor involvement in the nicotine's role on γ oscillations

**Figure 5:**
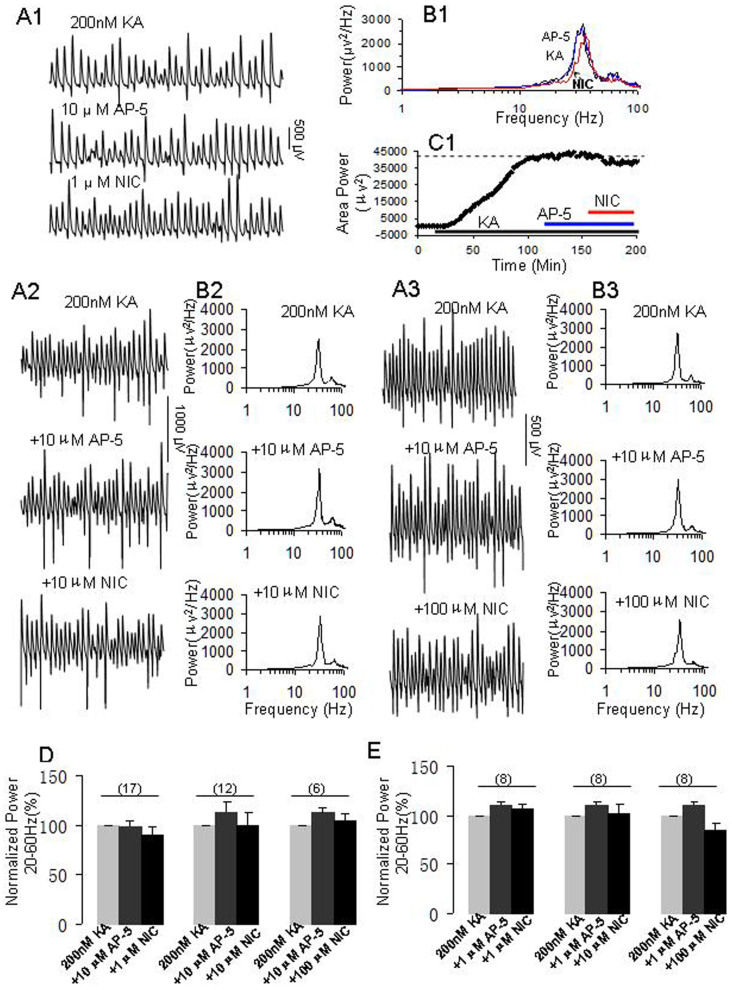
NMDA receptor antagonists, D-AP5 blocked the role of nicotine on γ oscillations. (A1–C1) The effects of 10 μM D-AP5 on 1 μM nicotine's role on γ. (A1): Representative extracellular recordings of field potentials in the presence of KA (200 nM) alone, KA + D-AP5 (10 μM) and KA + D-AP5 + NIC (1 μM). (B1): The power spectra of field potentials corresponding to the conditions shown in A1. (C1): Time course shows the changes in γ power before and after application of NIC in the presence of D-AP5. A2-B2: The effects of 10 μM D-AP5 on 10 μM nicotine's role on γ. (A2): Representative extracellular recordings of field potentials in the presence of KA alone, KA + D-AP5 (10 μM) and KA + D-AP5 + NIC (10 μM). (B2): The power spectra of field potentials corresponding to the conditions shown in A2. (A3–B3) The effects of 10 μM AP5 on 100 μM nicotine's role on γ. (A3): Representative extracellular recordings of field potentials in the presence of KA, KA + D-AP5 (10 μM) and KA + D-AP5 + NIC (100 μM). (B3): The power spectra of field potentials corresponding to the conditions shown in A3. (D): The bar graph summarizes the percent changes in γ power before (gray bars) and after various concentrations of nicotine (1–100 μM) in the presence of 10 μM D-AP5. 10 μM D-AP5 had no effect on γ oscillations (shallow dark bars) and the subsequent application of 1 μM nicotine had no significant effect on γ power (n = 17, black bars). 10 μM D-AP5 also blocked the roles of higher concentrations of nicotine (10 μM, n = 12; 100 μM, n = 6) on γ power. (E): The bar graph summarizes the percent changes in γ power before and after various concentrations of nicotine (1–100 μM) in the presence of 1 μM D-AP5. 1 μM D-AP5 had no effect on γ oscillations (shallow dark bars) and the subsequent application of 1 μM nicotine had no significant effect on γ power (n = 8, black bars). Similarly, 1 μM D-AP5 also blocked the roles of nicotine at higher concentrations of 10 μM (n = 8) and 100 μM (n = 8) on γ power.

Previous studies indicate that nAChR activation enhanced NMDA receptor function in the hippocampus^31^ and dorsolateral prefrontal cortex^33^. We have thus tested whether NMDA receptor activation contributes to the roles of nicotine on γ. When γ oscillations reached a steady state, NMDA receptor antagonist, D-AP5 (10 μM) was perfused for 40 min and no significant change on γ powers was observed, further application of nicotine (1 μM) caused no obvious changes on γ power (Fig. 5A1–C1). On average, the percent changes of γ powers were 100%, 98.8 ± 5.2% and 90.4 ± 7.6% for the control (KA alone), D-AP5 and D-AP5+nicotine, respectively. There was no statistically significant difference in γ powers between control and D-AP5 or D-AP5+nicotine (n = 17, *p > 0.05*, one way RM ANOVA).

Above results indicate that D-AP5 prevented nicotine-mediated enhancement of γ. We further tested whether D-AP5 was able to block the role of nicotine at higher concentrations on γ oscillation. 10 μM D-AP5 itself had no significant effect on γ oscillation, but completely blocked the enhancing role of 10 μM nicotine on γ power (n = 12, *p > 0.05*, one way RM ANOVA, Fig. 5A2, B2, D). Interestingly, 10 μM D-AP5 also blocked the suppression role of 100 μM nicotine on γ power (n = 6, *p > 0.05*, one way RM ANOVA, Fig. 5A3, B3, D).

D-AP5 (10 μM) itself had no effect on the peak frequency of oscillation (32.6 ± 1.3 Hz versus control 32.5 ± 1.0 Hz, n = 12), further application of nicotine (10 μM) did no change the peak frequency (32.8 ± 1.2 Hz versus 32.5 ± 1.0 Hz, n = 12). In another set of experiments, D-AP5 (10 μM) had no effect on peak frequency of oscillatory activity (29.4 ± 1.3 Hz versus control 29.9 ± 1.4 Hz, n = 6), further application of 100 μM nicotine decreased slightly the peak frequency (28.7 ± 1.5 Hz, *p > 0.05*, compared with D-AP5 treatment, n = 6).

Moreover, we tested the effects of a low concentration of D-AP5 (1 μM) on various concentrations of nicotine's role on γ. Our results showed that at such a low concentration, D-AP5 was able to block the enhancing role of nicotine (1–10 μM) (n = 8, Fig. 5E) and the suppression effect of nicotine (100 μM) on γ oscillations (n = 8, Fig. 5E). These results indicate that both the enhancing and suppressing effects of nicotine on γ oscillations involves NMDA receptor activation.

## Discussion

In this study, we demonstrated that nicotine at low concentrations enhanced γ oscillations in CA3 area of hippocampal slice preparation. The enhancing effect of nicotine was blocked by pre-treatment of a combination of α7 and α4β2 nAChR antagonists and by NMDA receptor antagonist. However,at a high concentration, nicotine reversely reduced γ oscillations, which can not be blocked by α4β2 and α7 nAChR antagonists but can be prevented by NMDA receptor antagonist. Our results indicate that nAChR activation modulates fast network oscillation involving in both nAChRs and NMDA receptors.

Nicotine induces theta oscillations in the CA3 area of the hippocampus via activations of local circuits of both GABAergic and glutamatergic neurons^13^,^38^ and is associated with membrane potential oscillations in theta frequency of GABAergic interneurons^39^. The modulation role of nicotine on γ oscillations may therefore involve in similar network mechanism as its role on theta.

In this study, the selective α7 or α4β2 nAChR agonist alone causes a relative small increment in γ oscillations, the combination of both agonists induce a large increase in γ oscillations (61%), which is close to the maximum effect of nicotine at 1 μM, suggesting that activation of two nAChRs are required to mimic nicotine' effect. These results are further supported by our observation that combined α4β2 and α7 nAChR antagonists, rather than either alone blocked the enhancing role of nicotine on γ. Our results indicate that both α7 and α4β2 nAChR activations contribute to nicotine-mediated enhancement on γ oscillation. These results are different from the previous reports that only a single nAChR subunit is involved in the role of nicotine on network oscillations. In tetanic stimulation evoked transient γ, α7 but not α4β2 nAChR is involved in nicotinic modulation of electrically evoked γ^40^; whereas α4β2 but not α7 nAChR is involved in auditory evoked γ oscillations *in vivo*^21^. The difference between the current study and others may be related to the difference in γ oscillatory model used or the way in γ induction. Pharmacologically induced γ are involved in excitatory and inhibitory synaptic transmission, while tetanic electrical stimulation-evoked γ involve only a pure inhibitory interneuron network^41^.

Our results are also different from the observation that nicotine at even 200 nM attenuats the carbachol-induced γ oscillations in the deep layers of rat prefrontal cortex (PFC)^42^. The local network difference between hippocampal CA3 area and prefrontal cortex may not be a factor to explain the different effect of nicotine on γ oscillations. A recent study by Acracri et al. (2010) has showed that nicotine decreases inhibitory postsynaptic potentials (IPSPs) rather than increases it when ionotropic glutamate receptors are blocked in the neurons of prefrontal cortex^19^. This study suggests that the role of nicotine on γ may be related to the status of ionotropic glutamate receptors or the level of glutamatergic tone and that a reduced tone of glutamatergic input may reverse the role of nicotine. In our study, KA-induced γ may have a higher level of glutamatergic tone than carbachol-induced γ, which may explain the different response of nicotine between two studies. This hypothesis, however, needs to be further tested.

Nicotine has been reported to regulate GABA release from interneurons such as perisomatic targeting parvalbumin-expressing cells via activation of nAChR located at presynaptic sites^43^, which may contribute to nicotine's enhancing role on γ oscillations.

NMDA receptor appears to be critically involved in both γ-enhancing and γ-suppressing effects of nicotine at low and high concentration, respectively. The involvement of NMDA receptor in nicotinic modulation of γ oscillations was supported by previous study that showed the activation of NMDA receptors on interneurons increased the frequency of cholinergically-induced γ oscillations in the mouse hippocampal CA3 region^44^. In this study, the NMDA receptor antagonists, D-AP5, had no obvious effect on KA-induced γ,which was in line with previous studies^34^,^45^. However, this result is different from the observation that acute application of ketamine, another NMDA receptor antagonist, increased KA-induced γ oscillations (but reduced the peak frequency)^29^, suggesting that different NMDA receptor antagonists may have differential roles in the modulation of γ oscillations.

Acute application of D-AP5 completely blocked the enhancing role of nicotine on γ, which was in line with the contributions of NMDA receptors to the nicotinic cholinergic excitation of CA1 interneurons in the rat hippocampus^46^ and the modulation of α7 nAChR on presynaptic NMDA receptor expression and structural plasticity of glutamatergic presynaptic boutons^47^ as well as the increment of γ oscillation in the hippocampal CA3 region by the activation of interneuronal NMDA receptors^44^.

The high concentration of nicotine reversely reduced γ oscillations, which can not be blocked by α4β2 and α7 nAChR antagonists but can be prevented by NMDA receptor antagonist. Our results are different from the study that showed nicotine at 100 μM enhanced tetanic-stimulation evoked transient γ^40^, the difference is likely explained by the different γ model used. Tetanic-stimulation evoked transient γ is only lasting a few seconds and the stimulation is far away from physiological condition. The compete blockage of down-regulation of nicotine on γ suggest that the role of nicotine at the 100 μM is a physiological response rather than non-specific action for such a concentration of nicotine. High concentration of nicotine may impose a rapid and strong NMDA receptor activation, causing a large calcium influx which negatively regulates γ oscillations. The reverse relationship between intracellular calcium and γ oscillations was demonstrated in previous studies^48^,^49^. It seems that at the high concentrations (10–100 μM), the activation of nAChRs and NMDA receptor play an opposite role on γ oscillations, as nAChR antagonists either exposed or worsen the effects of the down-regulation of nicotine at higher concentrations. Interestingly, it seems all concentrations of nicotine used in this study are able to activate NMDA receptors, as NMDA receptor antagonist at even a low concentration can block the different response of nicotine at various concentrations tested. Nevertheless, this study demonstrates the dose-dependent modulation of nicotine on γ oscillations and suggests that nAChR agonists may have a therapeutic effect in neuro-psychological disorders^24^.

### Clinical significance

The modulation of nicotine at different concentrations on γ oscillations and NMDA receptor function suggests that nAChR activation may be useful for the therapeutic application in schizophrenia, as the abnormal γ synchrony was demonstrated in the human^50^,^51^,^52^ and in the animal models^29^,^30^,^53^.

## Author Contributions

C.B.L. designed the experiment; Y.W., Z.W., J.G.W., X.Z., X.F.W. performed the experiments; C.B.L., Y.W. and Y.L.W. wrote the manuscript; C.B.L., Y.W., Z.W., J.S. and Z.H. analyzed the data. All authors reviewed the manuscript.
